# Climate and Land‐Use Change May Reshape the Biogeography of Freshwater Crabs Across China

**DOI:** 10.1002/ece3.73505

**Published:** 2026-04-29

**Authors:** Yiting Geng, Collins Oduro, Juanjuan Chen, Sangar Khan, Joyceline Adom Frimpong, Tatenda Dalu, Naicheng Wu

**Affiliations:** ^1^ School of Geography and Remote Sensing Ningbo University Ningbo China; ^2^ Zhejiang‐Germany Joint Laboratory on Remote Sensing of Coastal Ecosystem Ningbo University Ningbo China; ^3^ Department of Civil and Environmental Engineering Howard University Washington DC USA; ^4^ Centre for Invasion Biology, School for Climate Studies Stellenbosch University Stellenbosch South Africa; ^5^ Department of Hydrology and Water Resources Management Kiel University Kiel Germany

**Keywords:** China, climate and land‐use change, freshwater crabs, species distribution models

## Abstract

Freshwater biodiversity is increasingly exposed to the synergistic effects of climate forcing and land‐use change, yet the regional responses of key invertebrate lineages remain poorly resolved. We employed ensemble species distribution models to assess how future climate–land‐use trajectories may reorganize the suitability patterns for two ecologically distinct freshwater crab families in China: the inland Potamidae and the coastal‐estuarine Sesarmidae. Utilizing georeferenced occurrences from 2014 to 2024 and seven bioclimatic and land‐use predictors, we developed AUC‐weighted ensembles of MaxEnt and Random Forest models (AUC 0.91–0.94; TSS 0.75–0.77). Current suitability is concentrated within the humid river basins and coastal systems of southern and eastern China. Potamidae distributions are primarily associated with macro‐scale thermal gradients, whereas Sesarmidae suitability reflects a strong interaction between climatic variables and coastal land‐use signatures. Future projections (SSP1‐2.6 and SSP5‐8.5) indicate a systematic increase in mean and median continuous suitability across both families, suggesting a transition toward a more bioclimatically permissive landscape. However, the threshold‐defined suitable area contracted sharply, particularly for Sesarmidae, demonstrating that future change is better characterized as a spatial redistribution from concentrated contemporary cores toward broader, more diffuse intermediate‐suitability envelopes. While Potamidae exhibits a northward and inland expansion of moderate suitability, Sesarmidae maintains a restricted association with coastal refugia despite broader regional permissiveness. These results indicate that global change may expand environmental envelopes without preserving stable core habitats, underscoring the need to distinguish broad suitability from high‐confidence refugia in freshwater biodiversity conservation.

## Graphical abstract

We employed ensemble species distribution models to investigate how climate and land‐use change will impact habitat suitability for two freshwater crab families (Potamidae and Sesarmidae) across China. Temperature emerged as the dominant driver, with cropland and urban land having strong, family‐specific effects on suitability. Future scenarios predict significant northward and inland gains for Potamidae, but more modest, coastal‐confined gains for Sesarmidae, highlighting contrasting range‐shift potential under global change.

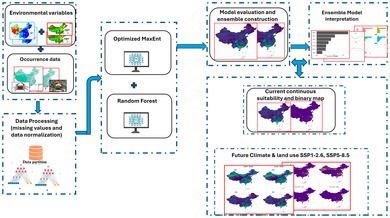

## 1 Introduction

Since the beginning of the 21st century, global climate change and rapid transformations in land‐use patterns have emerged as dominant drivers of ecosystem structure and function (Foley et al. 2005; Pereira et al. 2012). Human activities have already altered more than one‐third of the Earth's terrestrial surface through agricultural expansion, urbanization, and infrastructure development, accelerating habitat degradation and biodiversity loss (Torres‐Romero et al. 2024). Continued greenhouse gas emissions are projected to raise the global mean temperature by approximately 1.5°C–4.5°C by 2100, accompanied by shifts in precipitation regimes and an increase in the frequency of extreme events (IPCC 2023). Freshwater ecosystems are among the most sensitive to these changes. However, they cover < 1% of the planet's surface; they support around 6% of described species and are experiencing disproportionate biodiversity declines (Dudgeon et al. 2006; Oduro et al. 2025). These pressures are increasingly reshaping the biogeography of freshwater taxa, altering where species can persist and how assemblages are organized in space.

Within freshwater biotas, decapod crustaceans, especially freshwater crabs, are key components of benthic macroinvertebrate communities. They contribute to detritus processing, sediment bioturbation, and energy transfer between aquatic and riparian food webs, thereby influencing ecosystem functioning at multiple scales (Shi et al. 2021). Many freshwater crab lineages exhibit high endemism, narrow ranges, and strong fidelity to particular drainage systems, reflecting limited dispersal, historical basin isolation, and complex diversification histories. Their life cycles are often fully independent of the sea, making them informative model taxa for studying complete freshwater adaptation, biogeographic isolation, and cave speciation, as well as for linking past and present‐day range dynamics (Fang et al. 2013; Shi, Hao, et al. 2025). Because they are relatively sedentary and sensitive to water quality and habitat connectivity, freshwater crabs also serve as useful indicators of freshwater ecosystem health (Wang et al. 2025).

Climate and land‐use change can affect freshwater crabs through multiple, interacting pathways. Rising water temperatures alter metabolic rates, growth, and development, while changes in precipitation and flow regimes modify hydrological connectivity, habitat stability, and, in some systems, salinity conditions (Hewitt 2000; Xiao et al. 2022). Concurrent land‐use conversion, particularly agricultural expansion, urbanization, and wetland reclamation, fragments habitats, degrades water quality, and reduces the availability of refugia (Liu et al. 2011; Michel et al. 2021). Land‐use and land‐cover change not only modify local habitat structure but also feed back on the climate system via altered surface energy balance, carbon cycling, and hydrology, thereby intensifying pressures on freshwater biota (Liu et al. 2011). Given their low dispersal capacity and strong association with specific drainage networks, freshwater crabs are likely to be particularly vulnerable to these combined effects.

A coastal dimension is added by mangrove and estuarine wetlands, which are highly productive intertidal forests at the land–sea interface, providing shoreline protection, carbon storage, and habitat for a diverse array of fauna (Kathiresan and Bingham 2001; Lee 2008). In mangrove systems worldwide, crabs of the family Sesarmidae often occur at high densities and act as major habitat engineers and consumers, strongly influencing litter breakdown, sediment structure, and nutrient fluxes (Xu 2020). Because sesarmid crabs are tightly tied to mangroves and estuarine wetlands, they respond directly to sea‐level rise, coastal development, and mangrove loss. They are therefore key indicator taxa for coastal wetland health under global change. Together, inland freshwater crabs and coastal sesarmids provide a powerful comparative system for examining how climate warming and land‐use change will reorganize the biogeography of freshwater decapods in both riverine and coastal landscapes.

China, a recognized hotspot of freshwater biodiversity, has warmed faster than the global average and has undergone intense land‐use transitions, placing dual pressures on freshwater habitats and their biota (He et al. 2018; Liu et al. 2022). China harbors the highest diversity of freshwater crabs globally, far exceeding that of other species‐rich countries such as Thailand and Colombia (Cumberlidge et al. 2009). This fauna is dominated by the family Potamidae, which accounts for more than 90% of Chinese freshwater crab species and exhibits high levels of endemism (Chu et al. 2018). Potamid crabs inhabit headwaters, mid‐reach rivers, and lakes, with diversity centers in the Yangtze and Pearl River basins and adjacent provinces, and only a few species extend into the Yellow River basin and further north (Chu et al. 2018; Pan et al. 2021). Along the coast, mangrove and estuarine wetlands in Guangxi, Guangdong, Fujian, Taiwan, and Hainan support diverse sesarmid assemblages that function as central ecosystem engineers (Lin 1997; Xu 2020). While taxonomic, phylogenetic, and historical biogeographical work on Chinese freshwater crabs has advanced rapidly, their potential future distributions under combined climate and land‐use change remain poorly quantified at national scales.

Recent work has begun to quantify the spatial structure of freshwater crab diversity and conservation gaps in China, identifying richness and endemism hotspots and linking these patterns to human pressures and broad climate–landscape gradients (Shi, Wang, et al. 2025). At finer phylogeographic scales, integrative studies combining genetic evidence with species distribution modeling have shown that Quaternary climate fluctuations and drainage evolution helped structure potamid lineages and refugia, leaving persistent imprints on contemporary distributions (Fang et al. 2015). More broadly, freshwater invertebrates are expected to be highly responsive to warming and hydrological change because physiological thermal constraints and fragmented catchments can limit dispersal, while climate forcing often interacts with land‐use intensification and other stressors (Woodward et al. 2010; Zeng and Yeo 2018). However, national‐scale assessments that jointly incorporate future climate and land‐use trajectories, and explicitly contrast inland river‐basin taxa with coastal–estuarine assemblages, remain scarce, limiting anticipatory conservation planning.

Building on these advances, we utilize species distribution models (SDMs) to assess how climate and land‐use trajectories may impact habitats for two ecologically distinct but complementary freshwater crab families: the inland river‐dwelling Potamidae and the coastal estuarine Sesarmidae. SDMs relate species occurrence data to environmental predictors to infer potential distributions and evaluate responses to alternative future scenarios (Elith and Leathwick 2009). In China, recent applications of MaxEnt modeling have successfully predicted climate‐driven habitat shifts for diverse taxonomic groups, including plants (Luo, Si, et al. 2025; Luo, Peng, et al. 2025). Building on these advances, we utilize an ensemble SDM framework that combines optimized Maximum Entropy (MaxEnt) and Random Forest (RF) models with harmonized climate data (WorldClim v2.1; BCC‐CSM2‐MR) and land‐use data (LUH2; CLCD), and we interpret model outputs using SHapley Additive exPlanations (SHAP) and permutation‐based variable importance. Specifically, we aim to: (1) quantify current (2014–2024) spatial distribution patterns of Potamidae and Sesarmidae across China's major river basins and coastal systems; (2) assess the relative influence of key climatic variables and land‐use types on their habitat suitability; and (3) project future changes in suitable habitat under contrasting low‐ and high‐emission climate and land‐use scenarios [Shared Socio‐economic Pathway (SSP) 1‐2.6 and SSP5‐8.5] for the 2030s (2021–2040) and 2050s (2041–2060). By providing national‐scale projections of freshwater crab habitat under combined climate and land‐use change, this study offers a framework for anticipating range shifts, identifying emerging refugia, and informing conservation strategies for freshwater decapods in China and similar rapidly changing regions worldwide.

## 2 Materials and Methods

### 2.1 Study Area

**FIGURE 1:**
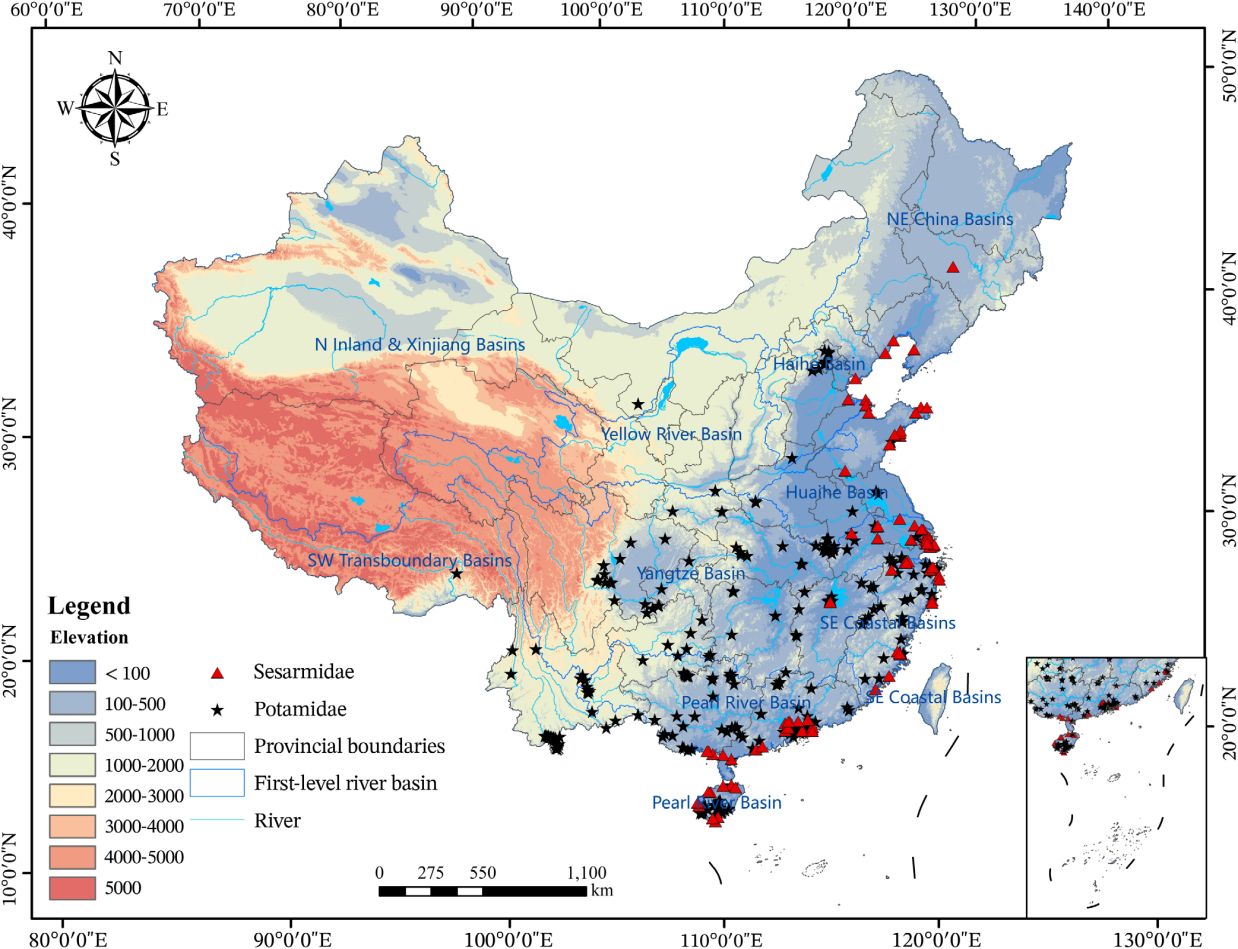
Study area and occurrence records used for species distribution models. Location of the study region within major river basins and coastal systems. Spatial distribution of Potamidae presence records. Spatial distribution of Sesarmidae presence records. Points show unique occurrences retained after spatial and environmental filtering.

This study was conducted within the territorial boundaries of mainland China, approximately spanning from 18° to 54° N in latitude and 73° to 135° E in longitude (Figure 1). Encompassing a vast land area of about 9.6 million km^2^, the region exhibits exceptional heterogeneity in topography, climate, and ecosystems (Wu, Dong, et al. 2025; Pereira et al. 2010), making it a significant global priority for biodiversity conservation and global change research (Zhao et al. 2022). China's hydrographic network is extensive and critical for aquatic biodiversity. Major river systems include the Yangtze River, Yellow River, Pearl River, and Heilongjiang River, along with numerous associated lakes and wetlands (Xing et al. 2020; Wang et al. 2021). These freshwater ecosystems range from high‐altitude glacial streams and alpine lakes on the Tibetan Plateau to large lowland rivers, floodplain lakes, and estuarine environments in the east. This diversity of aquatic habitats supports a rich community of aquatic macroinvertebrates, which play indispensable roles in ecosystem functioning (Dudgeon et al. 2006; Nash et al. 2016).

Climatically, the region spans multiple zones from a cold‐temperate climate in the north to a tropical climate in the south, influenced by the East Asian and Indian monsoons (Fick and Hijmans 2017). This results in strong gradients in mean annual temperature (from below −5°C in the northeast to above 20°C in the south) and annual precipitation (from less than 50 mm in the northwestern deserts to over 2000 mm in the southeastern coastal areas) (Fick and Hijmans 2017). These pronounced environmental gradients are known to be key determinants of species distributions at broad scales (Elith and Leathwick 2009; Pearson and Dawson 2003).

Comprehensive national‐scale studies across these diverse ecological regions are essential for understanding macroecological patterns and predicting responses to environmental change (Pereira et al. 2012, 2010). Selecting the entire country as the study area enables a systematic assessment of how freshwater macroinvertebrate distributions are shaped by and may respond to the interacting effects of climate and land‐use change across significant environmental gradients.

### 2.2 Species Occurrence Data

Distribution data for freshwater crabs served as the basis for this study, with a focus on two families: Sesarmidae, typically inhabiting mangrove and estuarine wetlands, and Potamidae, which are widely distributed in inland freshwater basins (Pan et al. 2021; Shi, Hao, et al. 2025). Primary species occurrence records (presence‐only) were obtained from the Global Biodiversity Information Facility (GBIF.org 2025; https://doi.org/10.15468/dl.frkhmq) using taxonomic name searches for all species within these families (GBIF.org 2025; Beck et al. 2014). Analysis was conducted at the family level to overcome the Wallacean shortfall associated with narrow‐range endemics, ensuring sufficient sample sizes for robust model calibration while assuming a degree of niche conservatism within these distinct ecological guilds (Wiens et al. 2010).

To ensure data quality and mitigate spatial bias, we implemented a rigorous cleaning protocol in line with established SDM practices (Aiello‐Lammens et al. 2015; Boria et al. 2014). First, duplicate records (identical coordinates and species) and records from areas outside mainland China or the oceans were removed after visual inspection. We then applied a temporal filter, retaining only records from 2014 to 2024 so that species occurrences reflect contemporary distributions. A total of 344 presence points were retained (254 for the Potamidae family and 90 for the Sesarmidae). Spatial autocorrelation was reduced by retaining only one occurrence record per 1 km × 1 km grid cell using the *spThin* R package (Aiello‐Lammens et al. 2015; Boria et al. 2014), thereby improving spatial independence for model calibration. The cleaned and thinned occurrences were subsequently overlaid with the environmental rasters and aggregated to the 0.1° modeling grid to match the resolution of the predictor layers used in the species distribution models.

### 2.3 Environmental Variables

Environmental predictors were selected to represent broad climatic and land‐use controls likely to influence freshwater crab distributions across China. In species distribution modeling, environmental variables are chosen to capture ecologically meaningful constraints on occurrence and habitat suitability rather than to maximize the number of predictors alone (Elith and Leathwick 2009). We therefore included climatic variables describing thermal and hydrological conditions, specifically annual mean temperature (BIO1), annual precipitation (BIO12), temperature seasonality (BIO4), and precipitation seasonality (BIO15), because temperature and water availability are fundamental determinants of freshwater decapod physiology, activity, survival, and habitat persistence (Gutiérrez and Beatty 2021; Toh et al. 2022). We also included cropland, pasture, and urban cover to represent major forms of anthropogenic landscape transformation that can alter freshwater and coastal habitats through runoff, sedimentation, hydrological modification, riparian disturbance, wetland loss, and shoreline change (Allan 2004). Full variable descriptions and data sources are provided in Table 1. To assess multicollinearity, we calculated pairwise Pearson correlations and variance inflation factors (VIF) for the retained variables. All VIF values were below 5 (1.11–4.78), indicating acceptable multicollinearity for model fitting (Table S2).

**TABLE 1:** Potential environmental factors affecting the distribution of the Potamidae and Sesarmidae families.

Environmental variables	Description	Unit
BIO1	Annual mean temperature	°C
BIO4	Temperature seasonality	°C
BIO12	Annual mean precipitation	mm
BIO15	Precipitation seasonality	mm
Urban	Urban land proportion	%
Crop	Cropland proportion	%
Pasture	Pasture proportion	%

To align the high‐resolution climate data (30 arc‐seconds) with the coarser land‐use (0.25°) and future climate projections (BCC‐CSM2‐MR), all layers were resampled to a consistent 0.1° modeling grid using bilinear interpolation. Land‐use predictors (Urban, Crop, and Pasture) were defined as the fractional proportions (%) of each grid cell occupied by each class. This characterizes the intensity of anthropogenic modification within the landscape matrix rather than as a discrete category at the occurrence point. Future land‐use variables were sourced from the LUH2 SSP‐RCP transitions (SSP1‐2.6 and SSP5‐8.5) for the 2021–2060 periods, ensuring temporal consistency across all future suitability projections.

From the LUH2 dataset, we selected the three land‐use classes representing the most severe and widespread anthropogenic pressures on freshwater ecosystems in China: cropland (agricultural pollution and alteration), urban (complete habitat loss and hydrological disruption), and pasture (riparian degradation and sedimentation). These represent direct, proximal threats to the water quality, substrate stability, and hydrological integrity that freshwater crabs require (Cumberlidge et al. 2009). This focused set of seven predictors yields a robust, interpretable model that captures the combined effects of core climate drivers and dominant human land use on habitat suitability. Current (1970–2000) bioclimatic variables were sourced from WorldClim version 2.1 at 30 arc‐second resolution (Fick and Hijmans 2017). To project future habitat suitability, we utilized climate projections from the BCC‐CSM2‐MR model (CMIP6) for the 2021–2040 and 2041–2060 periods under SSP1‐2.6 and SSP5‐8.5 scenarios (Riahi et al. 2017). All raster layers were processed to a consistent spatial resolution and coordinate system for analysis.

### 2.4 Species Distribution Modeling

We modeled the contemporary climatic suitability and potential distribution of Potamidae and Sesarmidae across China using a species distribution model (SDM) (Elith and Leathwick 2009). The response variable was a binary presence–absence indicator for each georeferenced record (1 = presence, 0 = pseudo‐absence). Because true absence data were unavailable, we generated pseudo‐absences by random sampling within the mainland China study extent, using a pseudo‐absence sample size equal to twice the number of presences for each family (1:2 presence:pseudo‐absence ratio). Pseudo‐absences were sampled randomly from the full mainland China modeling extent defined by the valid predictor grid for the Potamidae family (i.e., cells with complete land‐use layers). For sesarmidae (coastal accessible‐area background), pseudo‐absences were sampled randomly from a coastal buffer background, defined as all grid cells within 200 km of the coastline. This background reflects the accessible environment for coastal or estuarine systems and reduces inflation of apparent performance arising from environmentally distant pseudo‐absences. This sampling ratio has been widely applied in SDM workflows and has been reported as a reliable configuration for model training in presence‐only contexts when absences are unavailable (Barbet‐Massin et al. 2012; Liu et al. 2013). Pseudo‐absence points were sampled independently for each family and were used consistently across algorithms to enable comparable model fitting and evaluation.

The single best model may not be the optimal prediction model. Under certain conditions, ensemble modeling is considered more accurate than a single model (Wu, Bouma, et al. 2025). Hence, we implemented two complementary presence–absence algorithms: (i) a Maxent model fitted with the maxnet implementation (Phillips et al. 2006; Phillips and Dudík 2008; Merow et al. 2013), and (ii) a Random Forest (RF) classifier (Breiman 2001; Cutler et al. 2007; Liaw and Wiener 2002). For Maxent, we used the default set of linear, quadratic, product, and hinge features to predict cloglog‐transformed suitability, which is expressed on a probability scale between 0 and 1. For RF, we fitted 500 trees with a default mtry equal to the square root of the number of predictors, and we used the predicted probability of presence (class “presence”) as the suitability score.

All modeling was done in R version 4.5.2 using the packages terra, raster, maxnet, randomForest, pROC, dplyr, and ggplot2. Raster calculations and projections were handled with terra to allow disk‐based processing of large predictor stacks. For each family, we extracted the values of all seven predictors at the locations of the presence and pseudo‐absence points from the baseline environmental stack. Records with missing predictor values were discarded. To evaluate predictive performance, we used stratified 5‐fold cross‐validation. Presence and pseudo‐absence records were split into five folds with equal representation of both classes; four folds were used for training, and the remaining fold for testing.

For each fold and each algorithm, we calculated the area under the receiver operating characteristic curve (AUC) (Fielding and Bell 1997; Fawcett 2006; Sofaer et al. 2019) and the True Skill Statistic (TSS) (Fielding and Bell 1997). TSS was computed from the ROC curve using the threshold that maximized Youden's J (sensitivity + specificity−1). We report the mean and standard deviation of AUC and TSS over the five folds for each algorithm and family. After cross‐validation, final Maxent and RF models for each family were trained on the full dataset.

**FIGURE 2:**
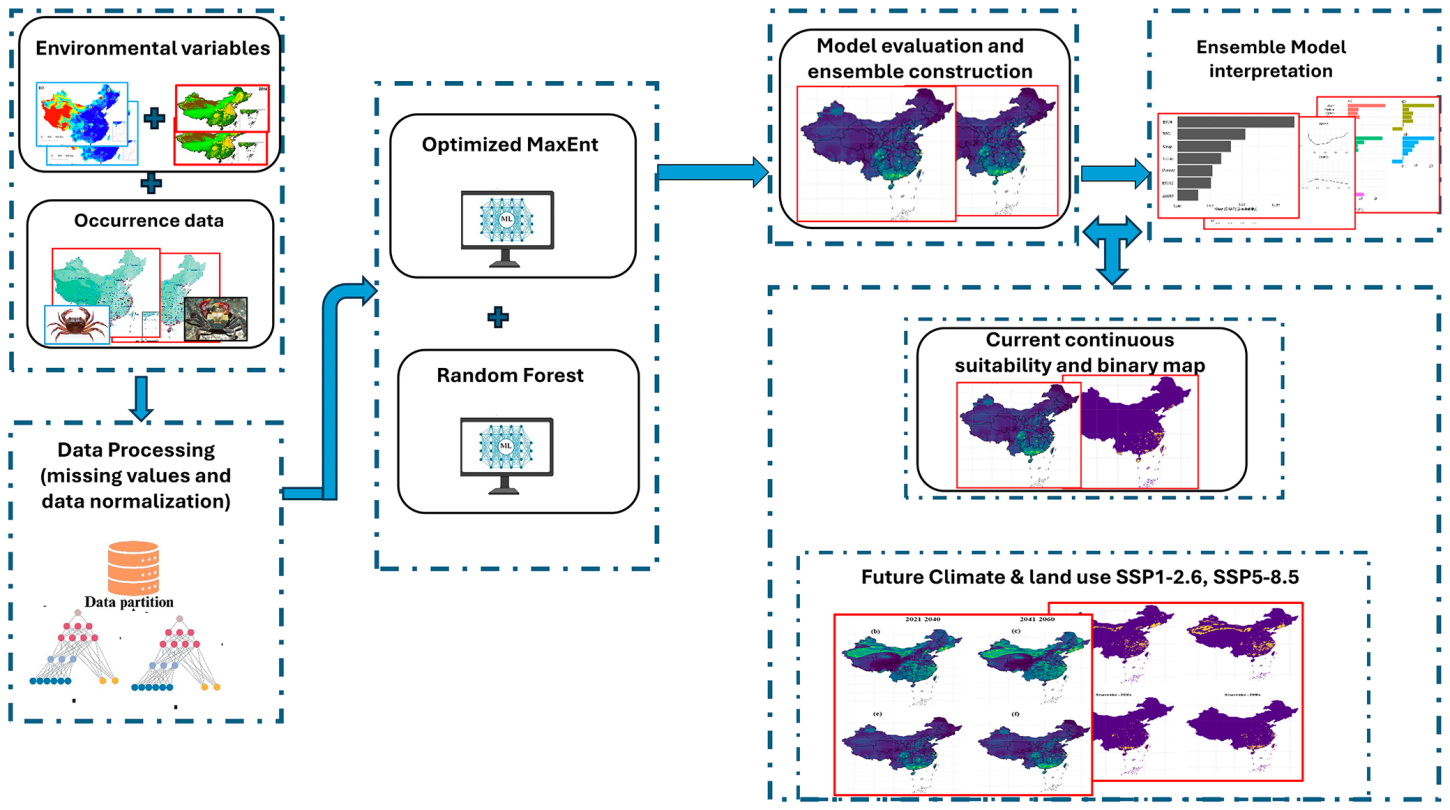
Workflow for ensemble species distribution modeling of freshwater crab habitat suitability in China.

To derive a conservative estimate of the current potential distribution, we converted continuous suitability predictions into binary presence–absence maps. For each fitted model, we first projected the model across the baseline environmental stack and extracted the predicted suitability at all known presence locations. The 10th percentile of these suitability values was then used as a presence threshold (P10) (Liu et al. 2005). This threshold corresponds to the suitability value above which 90% of the calibration presences are correctly classified as suitable (Ye et al. 2020). The baseline probability map was reclassified to presence (1) where suitability ≥ P10 and absence (0) otherwise. This binary map, combined with the cell‐area raster, allowed us to estimate the areal extent (km^2^) of climatically suitable habitat for each family under current conditions. The entire workflow has been shown in Figure 2.

#### 2.4.1 Ensemble Modeling and Projection of Suitability

To guard against overfitting and ensure our ensemble was based on models with strong predictive generalization, we used performance metrics derived from stratified 5‐fold cross‐validation. Because Maxent and RF often capture different aspects of species–environment relationships, we also constructed an AUC‐weighted ensemble for each family, following the principles of ensemble forecasting (Araújo and New 2007; Thuiller et al. 2009). Algorithms were retained in the ensemble only when they achieved AUC ≥ 0.90 and TSS ≥ 0.70, thereby serving as a conservative performance filter within a standard threshold‐based ensemble SDM framework (Thuiller et al. 2009). For Potamidae and Sesarmidae, both Maxent and RF met these criteria. Continuous ensemble suitability for each grid cell was calculated as the weighted mean of the Maxent and RF probabilities, with weights proportional to their respective mean cross‐validated AUC values. We then recalculated a P10 threshold from the ensemble suitability at presence locations and applied it to the ensemble baseline map to obtain ensemble‐based binary current distributions and area estimates. The same fitted MaxEnt, RF, and AUC‐weighted ensemble models were then projected onto each future climate–land‐use raster stack (SSP1‐2.6 and SSP5‐8.5 for 2021–2040 and 2041–2060) to generate scenario‐specific continuous suitability maps, from which we derived P10‐based binary maps and area estimates.

We summarize changes in continuous suitability across scenarios and calculated the mean, median, and 95th percentile of ensemble suitability values across all grid cells in the modeling domain for each family and scenario. The mean represents the average suitability across the landscape, the median reflects the central tendency of the suitability distribution and is less influenced by extreme hotspot cells, and the 95th percentile summarizes the upper tail of predicted suitability while avoiding over‐reliance on single‐cell maxima. These statistics were used to compare broad changes in the suitability surface under current and future climate–land‐use conditions (Araújo and New 2007).

#### 2.4.2 Model Interpretation

In order to interpret the fitted models and identify the main environmental drivers of habitat suitability, we combined permutation‐based variable importance, SHAP (SHapley Additive exPlanations) values, and partial dependence plots. We quantified the relative influence of predictors on the fitted models and computed permutation‐based variable importance. For each algorithm, each predictor was permuted several times in turn, and the resulting drop in AUC (ΔAUC) relative to the unpermuted model was averaged across repetitions. Larger ΔAUC values indicate a greater contribution of that predictor to discrimination. We also generated one‐dimensional partial dependence curves by varying one predictor over its observed range while holding the others at their median values, providing a visual summary of the shape and direction of each species–environment relationship.

Additionally, to evaluate the direct influence of variables on the probability scale and to interpret the AUC‐weighted ensemble models, we calculated SHAP values for each predictor using the fastshap package, following the SHAP framework proposed by Lundberg and Lee (2017). In our case, model outputs are suitability scores on a 0–1 scale, allowing SHAP values to be interpreted as the change in predicted suitability attributable to a given predictor at a specific location, conditional on the other predictors. For each predictor, we summarized global importance as the mean absolute SHAP value across all evaluation points. A higher mean SHAP therefore indicates a predictor that, on average, moves suitability predictions more strongly along the 0–1 scale. For interpretive clarity, we classified predictors as having strong (mean SHAP ≥ 0.05), moderate (0.02–0.05), or weak (< 0.02) effects. These thresholds are study‐specific and intended solely to facilitate comparison of predictors within this modeling framework; they are not equivalent to percentage contributions, explained variances, or variable importance metrics.

Moreover, we examined the functional form and direction of species–environment relationships using one‐dimensional partial dependence plots (PDPs) for each predictor. For a focal predictor, we varied its value over the observed range while holding all other predictors at their median values. We averaged the resulting predictions across all presence and pseudo‐absence points. These PDPs provide an intuitive visual summary of how changes in each climatic or land‐use variable affect predicted suitability for each family.

All suitability maps (baseline and future scenarios) were plotted using a common 0–1 sequential color scale, ensuring colors are directly comparable across families, algorithms, and scenarios. Binary maps were generated from ensemble suitability using a fixed two‐color scheme (unsuitable vs. suitable). For each family and algorithm, we exported the underlying GeoTIFF rasters for both continuous and binary outputs; these form the basis for all descriptions of present‐day and projected potential distributions of Potamidae and Sesarmidae across China.

## 3 Results

### 3.1 Spatial Distribution of Freshwater Crab Occurrences

The species occurrence data revealed a distinct and complementary biogeographic pattern for the two crab families (Figure 1). The Potamidae family, representing inland freshwater crabs, was widely recorded across the humid monsoon river basins of eastern and southern China. Records were concentrated in the Yangtze, Pearl, Southeast Coastal, and East Coastal basins, extending inland along major river networks. In contrast, the Sesarmidae family, which is associated with coastal wetlands, exhibits a highly restricted distribution, with almost all records clustered in the coastal provinces from Zhejiang to Hainan, particularly in the Pearl River Delta (Table S1). Critically, no occurrence records were obtained for either family in the arid and high‐elevation regions of northwestern China, including the Xinjiang basins.

### 3.2 Model Performance

Both modeling algorithms demonstrated strong discrimination between the two freshwater crab families. For Potamidae, Maxnet achieved a mean AUC of 0.93 ± 0.01 and a mean TSS of 0.75 ± 0.03, while Random Forest performed slightly better, with a mean AUC of 0.94 ± 0.01 and a mean TSS of 0.76 ± 0.02. Sesarmidae models also performed well, with Maxnet yielding a mean AUC of 0.91 ± 0.05 and a mean TSS of 0.76 ± 0.08, and Random Forest yielding a mean AUC of 0.92 ± 0.05 and a mean TSS of 0.77 ± 0.15 (Table 2). Because model performance was very similar between Maxnet and Random Forest, the AUC‐weighted ensemble assigned nearly equal contributions to both algorithms for each family. Thus, the final ensemble predictions reflect a balanced contribution of the two models rather than dominance by a single algorithm.

**TABLE 2:** Cross‐validated model performance (AUC and TSS) for Maxent and Random Forest for each taxon.

Family	AUC maxnet (mean ± SD)	TSS maxnet (mean ± SD)	AUC RF (mean ± SD)	TSS RF (mean ± SD)	P10 maxnet	P10 RF	P10 ensemble
Potamidae	0.93 ± 0.01	0.75 ± 0.03	0.94 ± 0.01	0.76 ± 0.02	0.50	0.78	0.64
Sesarmidae	0.91 ± 0.05	0.76 ± 0.08	0.92 ± 0.05	0.77 ± 0.14	0.48	0.75	0.68

Abbreviations: AUC, area under the receiver operating characteristic curve; P10, 10th percentile training‐presence threshold; TSS, true skill statistic.

The ensemble P10 thresholds were 0.64 for Potamidae and 0.68 for Sesarmidae (Table 2). These values were higher than the Maxnet‐specific thresholds and lower than the Random Forest‐specific thresholds, reflecting the ensemble predictions' intermediate position. The relatively high ensemble thresholds indicate that binary suitable‐area estimates are sensitive to the spatial distribution of suitability values and should therefore be interpreted alongside continuous suitability metrics.

### 3.3 Relative Influence and Environmental Drivers

To identify and interpret the key climatic and anthropogenic drivers of current habitat suitability, we employed a model‐agnostic interpretation framework. We first quantified the relative contribution and directional influence of each predictor using SHAP values. We then visualized the functional form of the relationship between the most influential predictors and the model's predicted suitability using PDPs. This approach identifies the environmental factors that most strongly influence the model's predictions and how changes in these factors impact potential suitability across their observed ranges.

**FIGURE 3:**
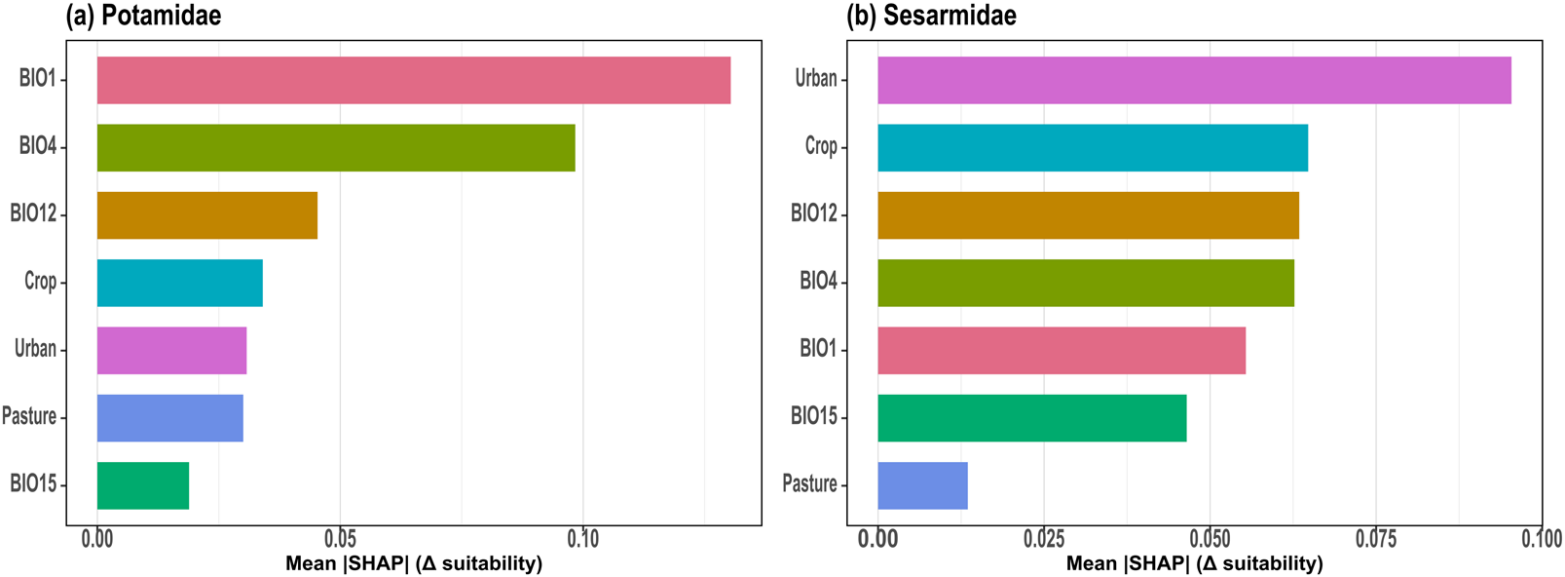
Mean absolute SHAP values for environmental predictors of ensemble habitat suitability for (a) Potamidae and (b) Sesarmidae.

Mean absolute SHAP values highlight the relative importance of environmental predictors, which differed substantially between families (Figure 3). For Potamidae (Figure 3a), the SHAP analysis identifies climatic factors as the dominant predictors, with BIO1 and BIO4 exerting the strongest relative influence, followed by BIO12. In contrast, the suitability of Sesarmidaee (Figure 3b) is primarily driven by anthropogenic land‐use variables, with urban cover as the most influential predictor, followed closely by crop cover and a combination of climatic variables (BIO12, BIO4, and BIO1).

**FIGURE 4:**
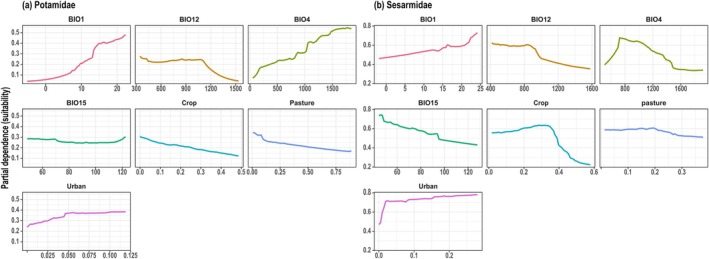
Partial dependence of ensemble habitat suitability on key climatic and land‐use predictors for (a) Potamidae and (b) Sesarmidae. Each panel shows the marginal effect of one predictor on predicted suitability (y‐axis, 0–1 scale) while holding all other variables at their mean values. The direction of the curve indicates the nature of the relationship: a positive slope signifies that suitability increases with the predictor, while a negative slope signifies that suitability decreases. The specificy‐axis value at any point represents the predicted suitability given that predictor's value, not an absolute probability of presence.

The PDPs revealed that Potamidae suitability increases sharply with higher BIO1 and BIO4, while showing a consistent decline as Crop and Pasture proportions increase (Figure 4a). Conversely, Sesarmidae suitability exhibits a strong, rapid positive response to urban cover and a non‐linear, hump‐shaped relationship with crop cover that peaks at moderate levels before declining (Figure 4b). While both families respond positively to BIO1, Sesarmidae suitability decreases with higher precipitation values (BIO12 and BIO15) and shows an intermediate peak for BIO4. All SHAP, PDP, and permutation‐importance results reported here are calculated from the ensemble models fitted to current conditions; these same models are then projected onto future climate–land‐use scenarios in Sections 3.4, 3.4.1, 3.4.2.

### 3.4 Current and Future Potential Habitat Suitability

#### 3.4.1 Current Habitat Suitability

**FIGURE 5:**
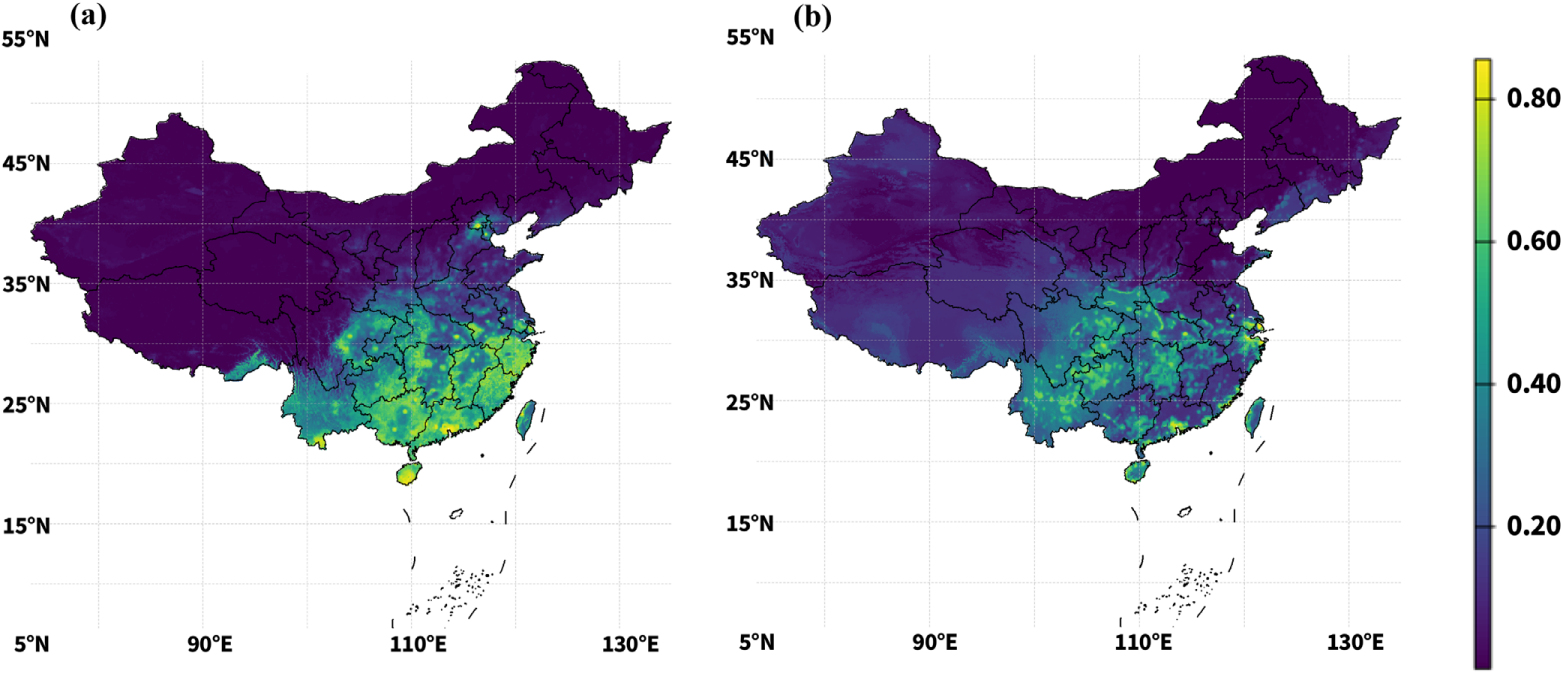
Current ensemble habitat suitability for freshwater crab families in China. (a) Potamidae (b) Sesarmidae.

Under current conditions, continuous ensemble predictions indicated that both freshwater crab families were concentrated primarily in southern and eastern China (Figure 5). Potamidae showed a broad southern distribution, with the highest suitability values extending across the Pearl River basin, the middle‐to‐lower Yangtze region, and southeastern China. Suitability declined toward northern and western China, with low predicted suitability across the Tibetan Plateau and the arid northwest. Sesarmidae displayed a more coastal and near‐coastal distribution, with the highest suitability concentrated in southeastern China, the lower Yangtze region, Hainan Island, the Pearl River delta, and nearby estuarine lowlands.

The continuous suitability statistics reflected these baseline patterns. For Potamidae, mean suitability was 0.14, whereas the median was only 0.01 (Table 3), indicating that highly suitable conditions were concentrated in relatively limited parts of the country while much of the domain remained weakly suitable. The area above fixed suitability thresholds was extensive, with 2,195,066 km^2^ above 0.3, 1,328,824 km^2^ above 0.5, and 397,330 km^2^ above 0.7. For Sesarmidae, the current mean and median suitability were 0.1457 and 0.0987, respectively, with 1,705,332 km^2^, 487,345 km^2^, and 69,472 km^2^ above the same fixed thresholds. The more conservative P10‐based binary areas (Figure S1) were substantially smaller, at 703,218 km^2^ for Potamidae and 96,406 km^2^ for Sesarmidae, indicating that binary core habitat occupied only a subset of the broader continuously suitable landscape.

**TABLE 3:** Mean, median, and 95th percentile of continuous ensemble suitability for Potamidae and Sesarmidae under current and future climate–land‐use scenarios.

Family	Scenario	Mean suitability	Median suitability	95th percentile
Potamidae	Current	0.14	0.01	0.67
Potamidae	SSP1‐2.6 (2021–2040)	0.20	0.18	0.47
Potamidae	SSP1‐2.6 (2041–2060)	0.24	0.22	0.53
Potamidae	SSP5‐8.5 (2021–2040)	0.20	0.17	0.49
Potamidae	SSP5‐8.5 (2041–2060)	0.22	0.18	0.54
Sesarmidae	Current	0.14	0.10	0.49
Sesarmidae	SSP1‐2.6 (2021–2040)	0.26	0.27	0.40
Sesarmidae	SSP1‐2.6 (2041–2060)	0.29	0.30	0.47
Sesarmidae	SSP5‐8.5 (2021–2040)	0.25	0.26	0.40
Sesarmidae	SSP5‐8.5 (2041–2060)	0.26	0.27	0.40

#### 3.4.2 Future Continuous Suitability Under SSP1‐2.6

**FIGURE 6:**
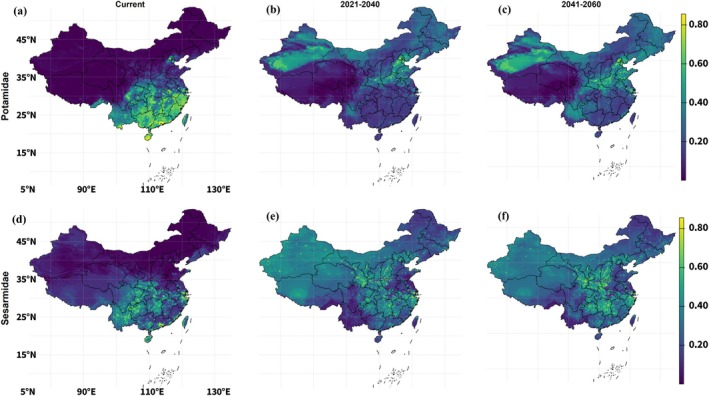
Current and future ensemble habitat suitability for Potamidae and Sesarmidae under the low‐emission scenario SSP1‐2.6. Panels (a–c) show suitability for Potamidae under current climate (a), 2021–2040 (b) and 2041–2060 (c). Panels (d–f) show suitability for Sesarmidae for the same periods. Colors indicate ensemble suitability (0–1), with warmer colors denoting higher predicted suitability.

Under SSP1‐2.6, both freshwater crab families showed clear increases in continuous habitat suitability, although the spatial patterns and magnitude of change differed between them (Figure 6). For Potamidae, future suitability extended beyond the current southern concentration into broader portions of central and northern China, particularly across inland transition zones and mid‐latitude basins. Both time windows, 2021–2040 and 2041–2060 experienced a change in core suitability areas and a marginal increase. However, the strongest increase was observed in the 2041–2060 period, when moderate suitability became more spatially extensive across interior and northern regions. The highest suitability values remained concentrated in southern China.

This pattern was reflected in the continuous suitability metrics (Table 3). Mean Potamidae suitability increased from 0.14 under current conditions to 0.20 in 2021–2040 and 0.24 in 2041–2060, while the median increased from 0.014 to 0.18 and 0.22, respectively. The strong rise in median suitability indicates that the increase was not restricted to a few isolated hotspots but reflected a broader upward shift across the suitability surface. The 95th percentile declined from 0.67 at present to 0.47 in 2021–2040, then increased slightly to 0.53 in 2041–2060, suggesting that while moderate suitability expanded widely, the upper end of suitability did not increase proportionally.

Sesarmidae showed an even stronger broadening of continuous suitability under SSP1‐2.6. The future maps indicated increasing suitability across large portions of eastern and central China, although the highest values remained associated with the southeastern and south‐central coastal belt. Mean suitability increased from 0.14 to 0.26 in 2021–2040 and 0.30 in 2041–2060, while median suitability rose from 0.10 to 0.27 and 0.30. As in Potamidae, the rise in the median indicates that suitability gains were distributed broadly across space rather than being confined to isolated cells. The 95th percentile decreased from 0.49 to 0.40 in 2021–2040 before increasing to 0.47 in 2041–2060, indicating that the upper tail of suitability remained comparatively stable but did not intensify to the same extent as the average suitability surface.

#### 3.4.3 Future Continuous Suitability Under SSP5‐8.5

**FIGURE 7:**
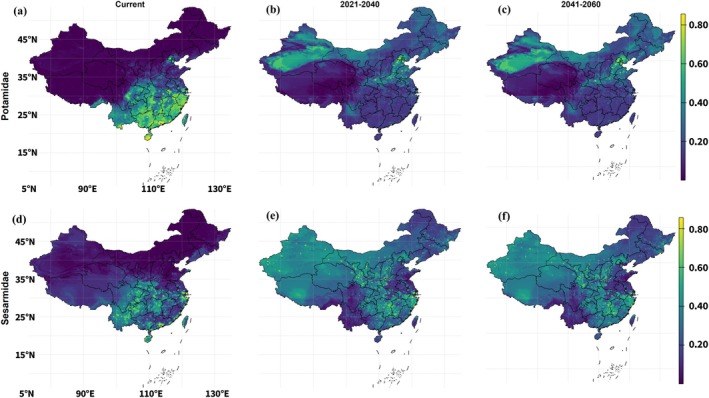
Current and future ensemble habitat suitability for Potamidae and Sesarmidae under the high‐emission scenario SSP5‐8.5. Panels (a–c) show suitability for Potamidae under current climate (a), 2021–2040 (b) and 2041–2060 (c). Panels (d–f) show suitability for Sesarmidae for the same periods. Colors indicate ensemble suitability (0–1), with warmer colors denoting higher predicted suitability.

The SSP5‐8.5 scenario produced a similar overall structure to SSP5‐2.6, but with some differences in magnitude and configuration (Figure 7). For Potamidae, continuous suitability again broadened inland and northward from the current southern concentration. Mean suitability increased to 0.20 in 2021–2040 and 0.22 in 2041–2060, while median suitability rose to 0.17 and 0.18, respectively, as shown in Table 3. The 95th percentile declined from 0.67 at present to 0.49 in 2021–2040 and then rose modestly to 0.53 in 2041–2060. Thus, although suitability became more widely distributed at moderate levels, the upper end of the distribution remained below the current baseline.

Sesarmidae showed a similarly pronounced increase in continuous suitability under SSP5‐8.5. Future maps indicated broader suitability across eastern and central China, with the strongest values still concentrated in southeastern and coastal lowlands. Mean suitability increased from 0.14 at present to 0.25 in 2021–2040 and 0.26 in 2041–2060, while median suitability rose from 0.10 to 0.26 and 0.27. As under SSP1‐2.6, this substantial increase in the median shows that suitability gains were broadly distributed across the landscape. The 95th percentile decreased from 0.49 at present to 0.40 in 2021–2040 and 0.41 in 2041–2060.

#### 3.4.4 Net Changes in Suitable Area Across Scenarios

When suitability was converted to binary habitat using the ensemble P10 threshold (Figures S1–S3), both freshwater crab families showed marked reductions in suitable area under all future scenarios (Table 4). For Potamidae, baseline binary suitable area was 703,218 km^2^, but declined to 46,370 km^2^ under SSP1‐2.6 in 2021–2040, 112,644 km^2^ under SSP1‐2.6 in 2041–2060, 38,856 km^2^ under SSP5‐8.5 in 2021–2040, and 137,371 km^2^ under SSP5‐8.5 in 2041–2060 (Table S3). These values correspond to net losses of approximately 656,848 km^2^, 590,574 km^2^, 664,362 km^2^, and 565,847 km^2^, respectively, relative to the current baseline.

**TABLE 4:** Current and projected area of suitable habitat (km^2^) for Potamidae and Sesarmidae under contrasting climate–land‐use scenarios (SSP1‐2.6 and SSP5‐8.5).

Family	Current km^2^	SSP1‐2.6 (2021–2040) km^2^	Δ SSP1‐2.6 (2021–2040) km^2^	SSP1‐2.6 (2041–2060) km^2^	Δ SSP1‐2.6 (2041–2060) km^2^	SSP5‐8.5 (2021–2040) km^2^	Δ SSP5‐8.5 (2021–2040) km^2^	SSP5‐8.5 (2041–2060) km^2^	Δ SSP5‐8.5 (2041–2060) km^2^
Potamidae	703,218	46,370	−656,848	112,644	−590,574	38,856	−664,362	137,371	−565,847
Sesarmidae	96,406	361	−96,045	21,345	−75,061	70	−96,336	328	−96,078

Sesarmidae showed an even stronger contraction under the same threshold‐based approach. Baseline binary suitable area was 96,406 km^2^ but declined to only 361 km^2^ under SSP1‐2.6 in 2021–2040, 21,345 km^2^ under SSP1‐2.6 in 2041–2060, 70 km^2^ under SSP5‐8.5 in 2021–2040, and 328 km^2^ under SSP5‐8.5 in 2041–2060. This represents net losses of approximately 96,045 km^2^, 75,061 km^2^, 96,336 km^2^, and 96,078 km^2^ (Table S3), respectively. Thus, under conservative binary thresholding, Sesarmidae retained only a very small fraction of its present suitable area in future projections.

## 4 Discussion

The ensemble models demonstrated high predictive performance for both Potamidae and Sesarmidae, indicating that the combined MaxEnt–Random Forest framework captured robust family‐level signals of freshwater crab habitat suitability across China (Hou et al. 2023). The current suitability maps show that both lineages remain concentrated in the warm, humid freshwater and coastal systems of southern and eastern China, with very low suitability across the arid northwest and the Tibetan Plateau. This spatial pattern is consistent with the known biogeographic concentration of Chinese freshwater crab diversity in subtropical and tropical river basins, estuaries, and coastal wetlands (Pan et al. 2021; Yeo et al. 2008).

Future projections suggest a complex redistribution of habitat rather than uniform range shifts (Warren and Seifert 2011). Under both SSP1‐2.6 and SSP5‐8.5 scenarios, we observed a systematic increase in mean and median continuous suitability across both families, reflecting a transition toward a more bioclimatically permissive landscape. Concurrently, however, the spatial extent of threshold‐defined suitable habitat contracted markedly, particularly under the conservative P10 threshold. This divergence reveals a fundamental decoupling in habitat dynamics: while moderate suitability becomes increasingly ubiquitous, high‐confidence “core” habitats fragment (Hole et al. 2011). Specifically, Potamidae exhibits a poleward expansion of its moderate‐suitability envelope northward and inland, consistent with documented latitudinal shifts for freshwater crustaceans under thermal forcing (Cumberlidge et al. 2009; Chu et al. 2018). Conversely, Sesarmidae displays a pervasive broadening of its suitability across eastern and central China while maintaining its coastal‐southeastern nexus, reflecting its specific sensitivity to modified coastal land use.

At broader regional and global scales, freshwater macroinvertebrates are recognized as sentinel taxa for climate change; warming modulates metabolic demand, phenology, and survival, while climate‐induced alterations to hydrologic regimes synergize with existing anthropogenic pressures such as land‐use change and pollution (Woodward et al. 2010; Liu et al. 2023). While long‐term monitoring shows that warming can radically restructure aquatic communities, the net balance between ecological “winners” and “losers” remains highly context‐dependent. For freshwater decapods, climate impacts are likely widespread, yet empirical modeling remains geographically uneven, particularly in high‐diversity hotspots (Toh et al. 2022). By resolving these patterns across China, our results demonstrate that future environmental forcing may broaden general permissiveness while simultaneously eroding the stable core habitats essential for long‐term persistence. These findings align with recent MaxEnt‐based assessments of climate change impacts on Chinese taxa, which have similarly identified complex patterns of habitat redistribution rather than simple range shifts (Luo, Si, et al. 2025; Luo, Peng, et al. 2025).

### 4.1 Interpretation of Key Drivers and Ecological Responses

The contrasting environmental controls identified for Potamidae and Sesarmidae reflect fundamental differences in their ecological niches and evolutionary constraints. For Potamidae, suitability is primarily dictated by thermal gradients (BIO1 and BIO4), reinforcing the role of temperature as the principal macro‐scale determinant for inland ectothermic macroinvertebrates (Urban et al. 2016). The positive association with warmer annual conditions and higher seasonality suggests that thermal energy availability limits the family's inland reach. However, this climatic template is significantly modified by land‐use pressures; the negative response to cropland and pasture suggests that agricultural conversion functions as a critical filter. These impacts likely manifest through a synergy of habitat fragmentation, altered hydrologic regimes, and agrochemical loading, stressors well‐documented to erode freshwater biodiversity (Allan 2004; Cumberlidge et al. 2009).

Sesarmidae suitability is dominated by anthropogenic land‐use signals, which appear to supersede individual climatic predictors. We interpret the strong positive correlation with urban cover not as biological affinity for built environments, but as a robust proxy for the complex geomorphological mosaic of the coastal‐estuarine interface. In China's coastal zones, urban infrastructure frequently co‐occurs with the essential habitat features required by sesarmids, such as tidal flats, mangrove remnants, and river mouths. Similarly, the non‐linear response to cropland implies that these taxa can persist within heterogeneous coastal matrices at low‐to‐moderate agricultural intensities, but reach a threshold of collapse under intensive landscape homogenization.

Ultimately, these results highlight that while climate defines the potential bioclimatic envelope, land‐use intensity dictates its functional availability. Cropland expansion emerges as a pervasive cross‐lineage stressor, though its mechanism varies. For Potamidae, it represents direct habitat degradation in inland lotic systems, and for Sesarmidae, it likely indicates degradation of estuarine water quality driven by upstream catchment modification. This highlights the need to integrate land‐use dynamics into species distribution models to avoid overestimating the availability of climate refugia in human‐dominated landscapes. Recent research by Petersen and Savini (2023) and Sofaer et al. (2019) confirms that climate‐only models trained on anthropogenically truncated occurrence data can significantly overestimate species' vulnerability, whereas hybrid models incorporating both climatic and land‐use variables produce more realistic projections.

### 4.2 Ecological Implications of Major Findings

Potamidae and Sesarmidae exhibit diverging suitability shifts that align with their distinct ecological niches and evolutionary constraints. Under future scenarios, Potamidae is associated with a northward expansion of bioclimatic permissiveness, a pattern consistent with a warming‐driven opening of thermal space in historically marginal high‐latitude regions. However, this expansion does not correspond to a gain in core habitat; rather, our results indicate a transition from concentrated southern strongholds to a broader, diffuse suitability structure. Equally, Sesarmidae maintains a tight spatial coupling with the coastal‐estuarine interface. While projections show a broadening of moderate suitability across eastern China, the family exhibits the most pronounced decline in threshold‐defined core habitat. This pattern is ecologically plausible for taxa associated with mangroves and tidal wetlands, where suitability reflects a synergy among macroclimate, shoreline architecture, salinity gradients, and tidal connectivity (Liu et al. 2011; Kuang et al. 2018; Bellard et al. 2013).

These contrasting responses illustrate a fundamental ecological principle that while inland taxa may gain suitability across extensive river networks, their realized occupancy remains physically constrained by basin structure and dispersal limitations. Meanwhile, coastal taxa may gain suitability in environmental space yet remain tethered to a shrinking set of intertidal refugia. Thus, future responses are better characterized as a spatial reorganization of suitability rather than a deterministic range shift. The observed increase in continuous suitability alongside binary area contraction is not contradictory; it signifies that future conditions favor broader zones of moderate permissiveness while reducing the density of high‐confidence core cells.

Finally, these projections represent potential suitability envelopes rather than forecasts of realized occupancy. Population persistence remains conditional on microhabitat specificity and unmodeled biotic interactions (Xiao et al. 2022). Furthermore, these patterns must be interpreted within the context of sampling effort. The paucity of contemporary records in northern China reflects the opportunistic nature of current data (e.g., GBIF) rather than confirmed biological absence (Beck et al. 2014). Consequently, the projected northern expansion identifies potentially permissive bioclimatic space that may already host undetected populations. Acknowledging this sampling‐suitability gap reframes our results as a strategic template for prioritizing northern basins for targeted field surveys, avoiding the overestimation of realized species' shifts in human‐dominated landscapes.

### 4.3 Implications for Conservation and Future Research

Potamidae management strategies should prioritize the preservation of contemporary high‐suitability strongholds while simultaneously enhancing longitudinal connectivity and headwater refugia in inland and northern basins. Our projections identify a significant expansion of bioclimatic permissiveness into several high‐latitude and high‐altitude river systems. Specifically, in Northwestern China, the Tarim, Ili, and Junggar basins emerge as priority regions where future thermal and precipitation regimes align with potamid environmental requirements. In the north and northeast, the Songhua, Liao, and Hai River basins also exhibit substantial increases in moderate‐suitability areas.

For Sesarmidae, the persistent concentration of high suitability along the southeastern seaboard underscores the urgency of protecting coastal wetlands and restoring mangroves. While regional suitability may expand, the most favorable environments remain tied to estuarine systems currently transitioning under pressure from land reclamation and shoreline hardening. Integrating sesarmid conservation into broader blue‐carbon initiatives and coastal resilience frameworks could yield significant co‐benefits for biodiversity and carbon sequestration (Mcleod et al. 2011).

Crucially, broad future suitability should not be conflated with successful range expansion. For habitat specialists with limited dispersal capacity, suitability maps serve better as a coarse‐scale filter for identifying priority monitoring zones and proactive conservation sites where environmental conditions may remain favorable. These projections also hold preliminary value for sustainable aquaculture planning by identifying basins and coastal segments likely to remain stable under future climate–land–use forcing. However, translating these surfaces into operational aquaculture management requires further integration of water quality parameters, salinity dynamics, and disease risk assessments (Khan et al. 2025).

A limitation of this study is that modeling was conducted at the family level, which may mask ecological differences among species within each lineage. This is particularly relevant for Sesarmidae, as not all species may be equally restricted to mangrove or estuarine habitats. The comparison presented here should therefore be interpreted as a broad family‐level contrast rather than a uniform ecological characterization of all constituent species. Species‐level modeling will be needed to assess the extent to which these lineage‐level patterns hold across individual taxa within Potamidae and Sesarmidae.

Future research should move toward multi‐model ensembles and finer‐resolution hydrologic data to better resolve the nuances of basin connectivity and coastal wetland persistence. Transitioning from family‐level analyses to species‐specific models will be vital for identifying particularly vulnerable lineages within the Potamidae and Sesarmidae. Furthermore, integrating physiological tolerances and life‐history traits into mechanistic models and linking them to socio‐economic development scenarios will provide a more realistic assessment of how anthropogenic interventions may mediate the impacts of global change on freshwater biodiversity.

## 5 Conclusion

This study employs an ensemble of optimized MaxEnt and RF models to characterize shifting suitability landscapes for two ecologically distinct freshwater crab families across China. Our analysis demonstrates that while Potamidae distributions are primarily structured by macro‐scale thermal gradients (annual mean temperature and seasonality), Sesarmidae suitability reflects a complex interaction between climatic variables and coastal land‐use signatures, where urban and agricultural cover serve as spatial proxies for estuarine geomorphology. Future projections under SSP1‐2.6 and SSP5‐8.5 scenarios reveal a fundamental spatial reorganization rather than a uniform range shift. While mean continuous suitability is projected to increase across both families, indicating broader environmental permissiveness, threshold‐defined core habitats undergo a sharp contraction. This manifests as an inland and northward expansion of the Potamidae suitability envelope, alongside severe fragmentation of the high‐confidence coastal strongholds essential to Sesarmidae.

These results imply that global change may broaden potential bioclimatic envelopes without preserving the stable, high‐quality habitats required for long‐term population persistence. Because these projections represent potential environmental space rather than deterministic forecasts of realized occupancy, they must be interpreted alongside constraints such as dispersal limitation, hydrological barriers, and fine‐scale habitat quality. Nevertheless, our findings identify urgent conservation priorities: the preservation of longitudinal river connectivity and southern freshwater refugia for Potamidae, and the safeguarding of estuarine and mangrove systems for Sesarmidae. Future research integrating physiological tolerances and socio‐economic drivers will be essential to translate these broad‐scale projections into robust, actionable strategies for conserving China's freshwater decapod diversity amidst rapid environmental change.

## Author Contributions

**Yiting Geng:** data curation (equal), formal analysis (equal), investigation (equal), methodology (equal), writing – original draft (equal), writing – review and editing (equal). **Collins Oduro:** conceptualization (equal), data curation (equal), formal analysis (equal), investigation (equal), methodology (equal), software (equal), visualization (equal), writing – original draft (equal), writing – review and editing (equal). **Juanjuan Chen:** data curation (equal), formal analysis (equal), methodology (equal), validation (equal), writing – original draft (equal), writing – review and editing (equal). **Sangar Khan:** data curation (equal), funding acquisition (equal), investigation (equal), methodology (equal), resources (equal), writing – review and editing (equal). **Joyceline Adom Frimpong:** data curation (equal), investigation (equal), software (equal), validation (equal), visualization (equal), writing – review and editing (equal). **Tatenda Dalu:** data curation (equal), investigation (equal), methodology (equal), project administration (equal), validation (equal), writing – review and editing (equal). **Naicheng Wu:** data curation (equal), funding acquisition (equal), investigation (equal), project administration (equal), resources (equal), supervision (equal), validation (equal), writing – original draft (equal), writing – review and editing (equal).

## Funding

This study was supported by grants from the National Natural Science Foundation of China (nos. 52279068, 42350410434).

## Conflicts of Interest

The authors declare no conflicts of interest.

## Supporting information


**Data S1:** Codes.

**Data S2:** README.

**Figure S1:** Current ensemble habitat binary suitability for freshwater crab families in China. (a) Potamidae (b) Sesarmidae.
**Figure S2:** Change in predicted binary suitable habitat area between baseline and future scenario. Spatial changes under SSP1‐2.6 and 2030s vs. 2050s.
**Figure S3:** Change in predicted suitable habitat area between baseline and future scenario. Spatial changes under SSP1–SSP5‐8.5 and 2030s vs. 2050s.
**Table S1:** Occurrence records used for species distribution models.
**Table S2:** Variance inflation factors (VIF) for the environmental predictors. All VIF values were below 5, indicating acceptable multicollinearity among predictors.
**Table S3:** Extended continuous suitability metrics and threshold habitat area for Potamidae and Sesarmidae under current and future scenarios.

## Data Availability

The occurrence data used in this study were sourced from the Global Biodiversity Information Facility (GBIF.org 2025; https://doi.org/10.15468/dl.frkhmq), and a complete list of these records is provided in Table S1. Climatic predictor variables (BIO1, BIO4, BIO12, BIO15) were obtained from WorldClim v2.1 and CMIP6 BCC‐CSM2‐MR projections under SSP1‐2.6 and SSP5‐8.5, while land‐use predictors (cropland, pasture, and urban proportion) were derived from the Land‐Use Harmonization 2 (LUH2). Additionally, the R scripts and code developed for the biomod2 species distribution modeling framework, including data preparation and analysis, are available as [Link], [Link], [Link] to ensure full reproducibility of our research.
