# Supplementary material for: Climate and Land‐Use Change May Reshape the Biogeography of Freshwater Crabs Across China
Source: Ecol Evol. 2026 Apr 29;16(5):e73505. doi: 10.1002/ece3.73505 (PMC13125959; doi:10.1002/ece3.73505)
Supplement: Supplementary file 3 — Figure S1: Current ensemble habitat binary suitability for freshwater crab families in China. (a) Potamidae (b) Sesarmidae. Figure S2: Change in predicted binary suitable habitat area between baseline and future scenario. Spatial changes under SSP1‐2.6 and 2030s vs. 2050s. Figure S3: Change in predicted suitable habitat area between baseline and future scenario. Spatial changes under SSP1–SSP5‐8.5 and 2030s vs. 2050s. Table S1: Occurrence records used for species distribution models. Table S2: Variance inflation factors (VIF) for the environmental predictors. All VIF values were below 5, indicating acceptable multicollinearity among predictors. Table S3: Extended continuous suitability metrics and threshold habitat area for Potamidae and Sesarmidae under current and future scenarios. [file ECE3-16-e73505-s002.docx]

**Supplementary material**

**Climate and land-use change may reshape the biogeography of freshwater crabs across China**

**Table S1. Occurrence records used for species distribution models**

| Distribution area  (Province) | Occurrence records | |
| --- | --- | --- |
|  | Sesarmidae | Potamidae |
| Zhejiang | 21 | 25 |
| Shanghai | 13 | 1 |
| Guangdong | 12 | 34 |
| Shandong | 10 | 1 |
| Hainan | 9 | 29 |
| Jiangsu | 7 | 4 |
| Fujian | 5 | 12 |
| Guangxi | 4 | 24 |
| Hebei | 3 | 0 |
| Liaoning | 3 | 0 |
| Anhui | 1 | 16 |
| Jiangxi | 1 | 9 |
| Jilin | 1 | 0 |
| Henan | 0 | 3 |
| Yunnan | 0 | 33 |
| Sichuan | 0 | 13 |
| Guizhou | 0 | 16 |
| Hubei | 0 | 12 |
| Beijing | 0 | 8 |
| Hunan | 0 | 6 |
| Chongqing | 0 | 4 |
| Shanxi | 0 | 2 |
| Xizang | 0 | 1 |
| Neimenggu | 0 | 1 |

Table S2. Variance inflation factors (VIF) for the environmental predictors. All VIF values were below 5, indicating acceptable multicollinearity among predictors.

| Variable | VIF |
| --- | --- |
| BIO4 | 4.78 |
| BIO1 | 3.58 |
| BIO12 | 2.29 |
| BIO15 | 2.0 |
| pasture | 1.88 |
| crop | 1.48 |
| urban | 1.11 |

Table S3. Extended continuous suitability metrics and threshold habitat area for Potamidae and Sesarmidae under current and future scenarios.

| **Family** | **Scenario** | **Minimum** | **Maximum** | **Area ≥ 0.3 (km²)** | **Area ≥ 0.5 (km²)** | **Area ≥ 0.7 (km²)** |
| --- | --- | --- | --- | --- | --- | --- |
| Potamidae | Current | 0.0000 | 0.9451 | 2,195,066 | 1,328,824 | 397,330 |
| Potamidae | SSP1-2.6 (2021–2040) | 0.0001 | 0.8802 | 2,103,788 | 309,395 | 12,318 |
| Potamidae | SSP1-2.6 (2041–2060) | 0.0001 | 0.8823 | 3,132,667 | 678,403 | 19,655 |
| Potamidae | SSP5-8.5 (2021–2040) | 0.0001 | 0.8835 | 1,932,003 | 420,063 | 11,851 |
| Potamidae | SSP5-8.5 (2041–2060) | 0.0004 | 0.9072 | 2,341,210 | 616,949 | 21,427 |
| Sesarmidae | Current | 0.0003 | 0.9241 | 1,705,332 | 487,345 | 69,472 |
| Sesarmidae | SSP1-2.6 (2021–2040) | 0.0042 | 0.7117 | 3,418,182 | 103,444 | 19 |
| Sesarmidae | SSP1-2.6 (2041–2060) | 0.0042 | 0.8029 | 4,890,720 | 379,298 | 11,499 |
| Sesarmidae | SSP5-8.5 (2021–2040) | 0.0056 | 0.6992 | 3,416,190 | 93,902 | 0 |
| Sesarmidae | SSP5-8.5 (2041–2060) | 0.0065 | 0.7116 | 3,615,381 | 77,616 | 52 |


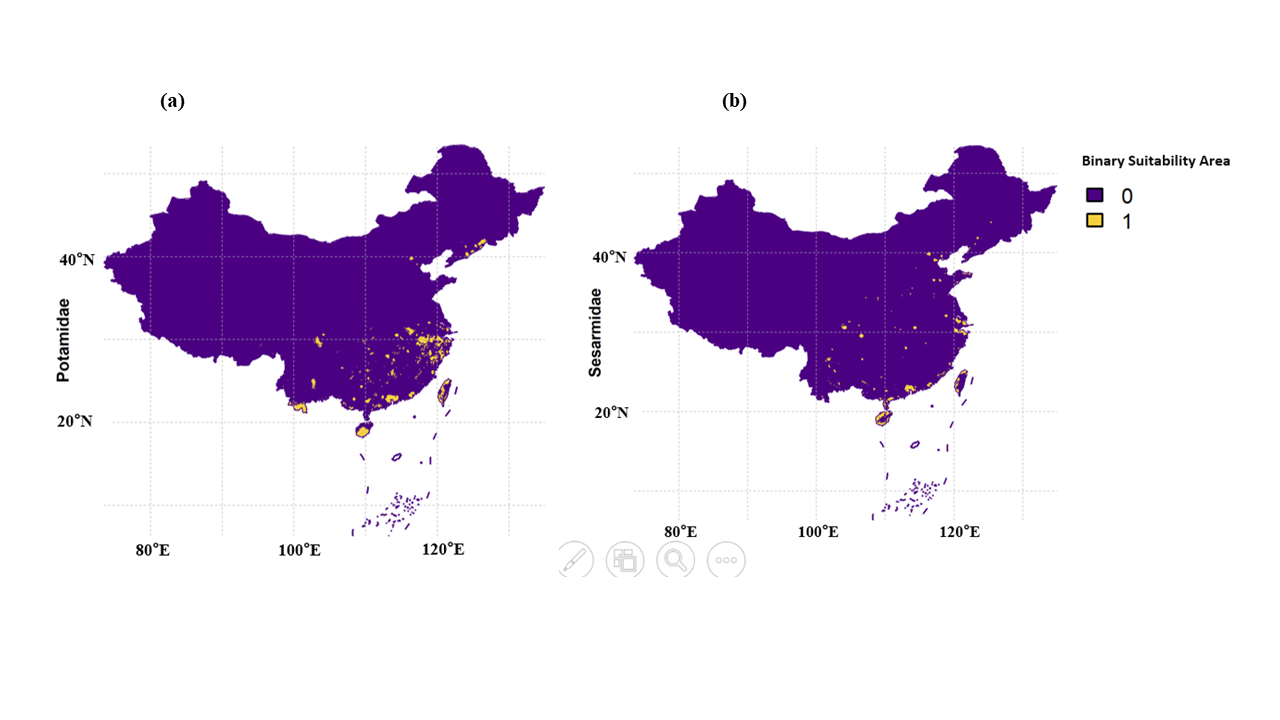


Figure S1: Current ensemble habitat binary suitability for freshwater crab families in China. (a) Potamidae (b) Sesarmidae.


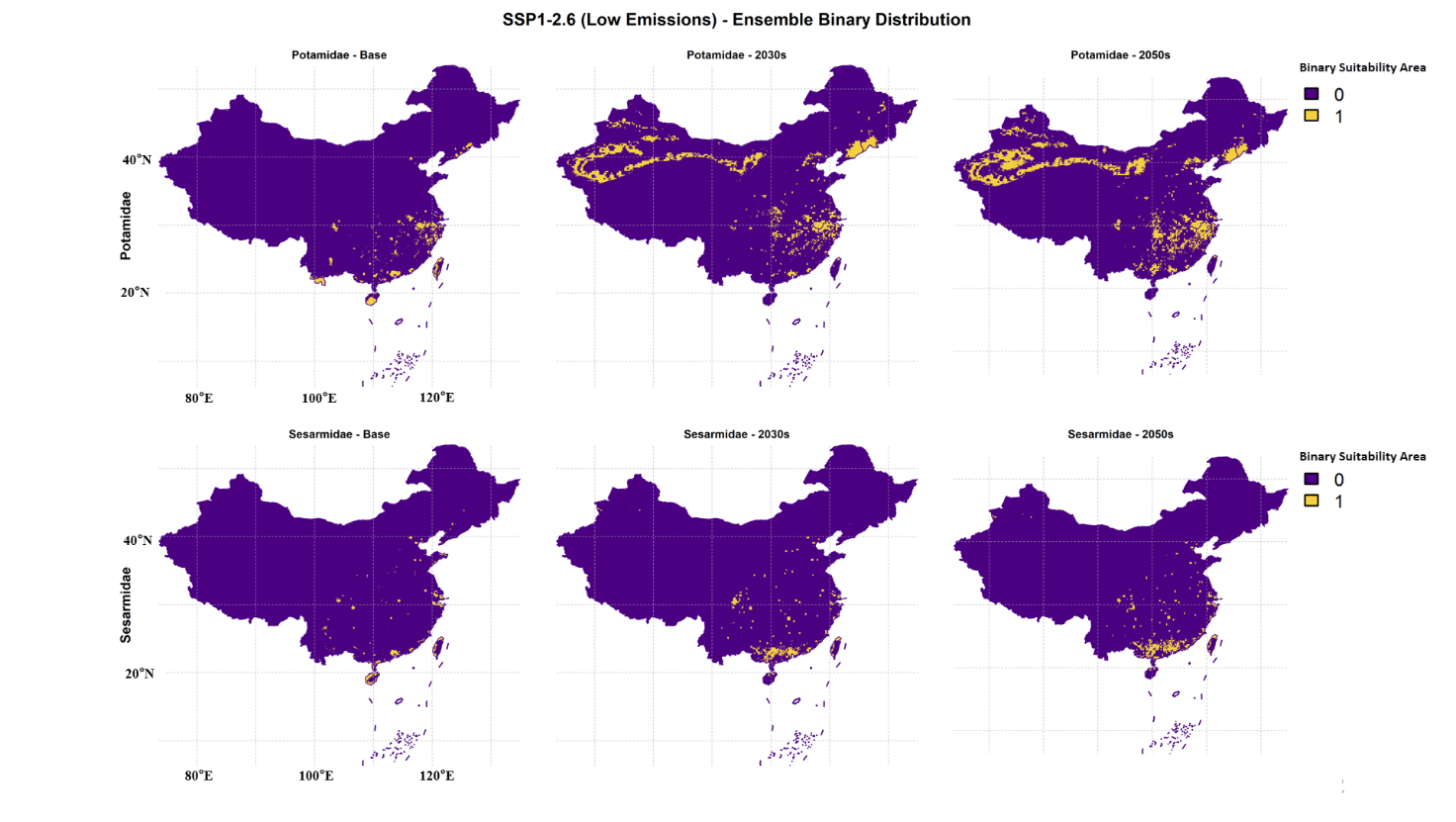


Figure S2: Change in predicted binary suitable habitat area between baseline and future scenario. Spatial changes under SSP1-2.6 and 2030s vs 2050s.


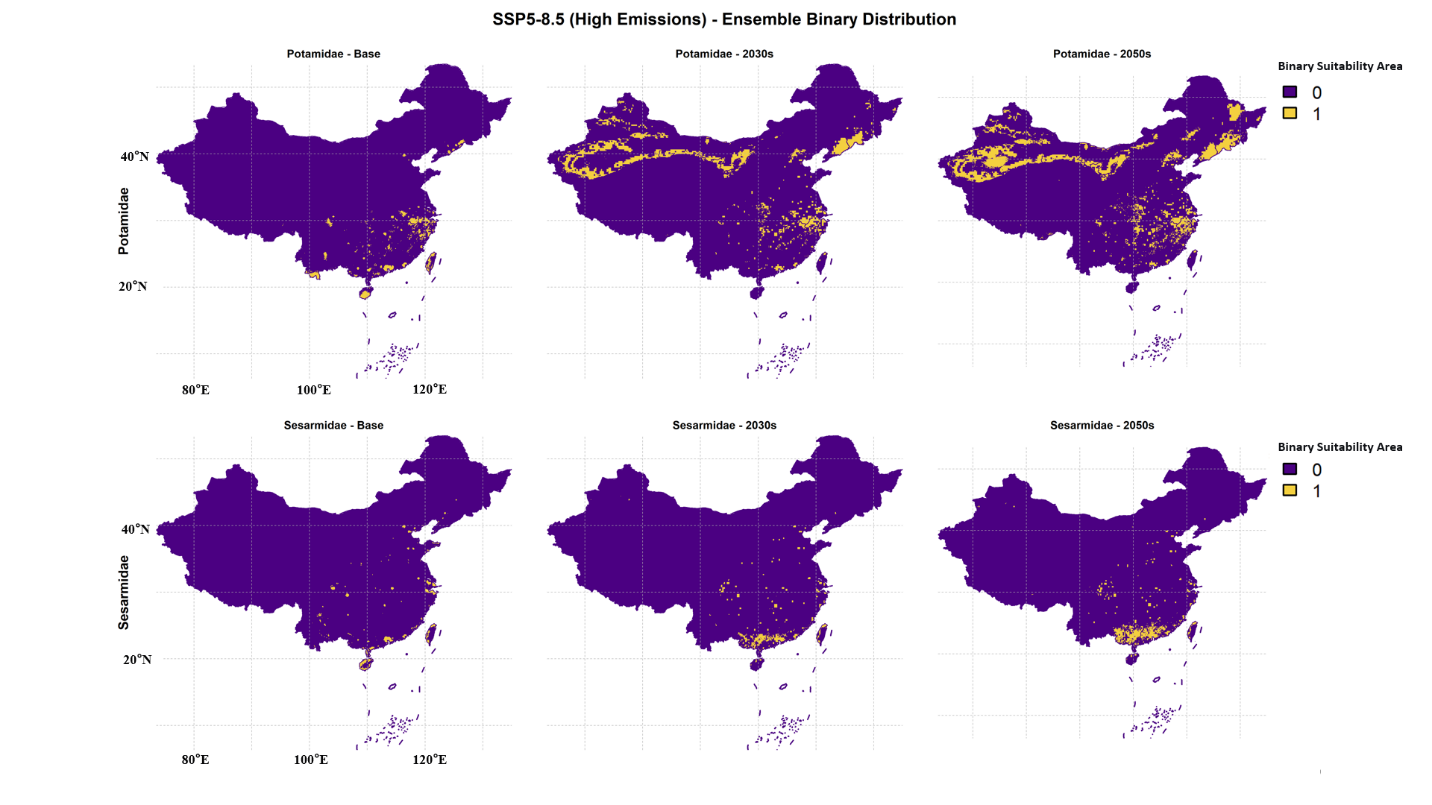


Figure S3 Change in predicted suitable habitat area between baseline and future scenario. Spatial changes under SSP1- SSP5-8.5 and 2030s vs 2050s.
